# Predicting the potential distribution in China of Euwallacea fornicatus (Eichhoff) under current and future climate conditions

**DOI:** 10.1038/s41598-017-01014-w

**Published:** 2017-04-19

**Authors:** Xuezhen Ge, Chao Jiang, Linghong Chen, Shuang Qiu, Yuxiang Zhao, Tao Wang, Shixiang Zong

**Affiliations:** 1 Key Laboratory of Beijing for the Control of Forest Pests, Beijing Forestry University, Beijing, 10083 P.R. China; 2 College of Forest Science, Beijing Forestry University, Beijing, 100083 P.R. China; 3 College of Environment and Resources, Jilin University, Changchun, 130012 P.R. China; 4 Department of Afforestation, National Forestry Bureau, Beijing, 100714 P.R. China; 5 Mentougou Forestry Station, Beijing, 102300 P.R. China

## Abstract

*Euwallacea fornicatus* (Eichhoff) is an important forest pest that has caused serious damage in America and Vietnam. In 2014, it attacked forests of *Acer trialatum* in the Yunnan province of China, creating concern in China’s Forestry Bureau. We used the CLIMEX model to predict and compare the potential distribution for *E. fornicates* in China under current (1981–2010) and projected climate conditions (2011–2040) using one scenario (RCP8.5) and one global climate model (GCM), CSIRO-Mk3-6-0. Under both current and future climate conditions, the model predicted *E. fornicates* to be mainly distributed in the south of China. Comparing distributions under both climate conditions showed that the area of potential distribution was projected to increase (mainly because of an increase in favourable habitat) and shift to the north. Our results help clarify the potential effect of climate change on the range of this forest pest and provide a reference and guide to facilitate its control in China.

## Introduction

The polyphagous shot-hole borer, *Euwallacea fornicatus* (Eichhoff) (Coleoptera: Curculionidae), is native to Southeast Asia and has been accidentally introduced to many countries throughout the world, including countries in Africa, Asia, and the Americas^1,2^. It is a selective, inconspicuous pest that attacks as many as 207 different tree species from 58 families, including *Camellia sinensis* (Theaceae), *Persea bombycina* and *P. Americana* (Lauraceae), *Acer buergerianum* (Aceraceae), and *Litchi chinensis* and *Dimocarpus longan* (Sapindaceae)^3^. Because it has so many kinds of host plants, damaging them both mechanically and through infection with symbiotic fungi, and spreads and reproduces rapidly, *E. fornicatus* has caused huge economic losses of million dollars in US and has had a serious environmental impact in parts of the US and Vietnam^1,4,5^. In China, *E. fornicates* has caused serious damage to economic tree species (*Litchi chinensis*, *Dimocarpus longan, Camellia* sp., etc) in Zhangzhou, Fujian and Tengchong, and Yunnan over many years. In 2014, the damage to street trees (*Acer buergerianum*, *Platanus acerifolia*, and *Paulownia* sp., etc.) caused by *E. fornicates* was considerable in Kunming, Yunnan. In 2015, *E. fornicates* was found in Hangzhou, Zhejiang^2,6,7^. The degree of infestation and the number of regions in China in which *E. fornicates* has been recorded are increasing. In view of the serious impact that *E. fornicates* has already caused, its further spread in China could cause huge damage to plantations of broadleaf trees. Nowadays, *E. fornicates* is considered a high-risk quarantine pest of international concern^1,4^.

Ambient temperature is one of the main factors that limits the distribution of ectothermic organisms such as insects. Anthropogenic climate change, which involves a rise in global temperatures, will thus have an important effect on the geographical distribution of insects^8^. In response to the changing climate, many species have already been observed to shift their distributions^9–12^. Depending on their climate tolerance, organisms will adapt to the new conditions in their native habitats, migrate to new habitats with appropriate climate conditions, or perish^13^.

The Fifth Assessment Report (AR5) of the Intergovernmental Panel on Climate Change (IPCC) clearly stated that the Earth’s climate is changing and that the trend of global warming is indisputable. Global temperatures are projected to increase by 0.3–4.8 °C by the end of the 21^st^ century^14^. With the progress that has been made in the development of climate models to simulate future climate scenarios, predicting changes in the potential geographic distribution of pest species has become a hot area of research^13,15–17^. A number of models are used to predict species distributions, including ANUCLIM/BIOCLIM, CLIMATE, CLIMEX, DOMAIN, GARP, HABITAT and MaxEnt^18^. Among them, CLIMEX software is considered a comprehensive and reliable inferential modelling software that can produce a niche model without requiring pseudo-absence data, take into account both the physiological data and known distribution data, and predict potential distribution. It also focuses more on species’ physiological characteristics than other models^19,20^. The CLIMEX model has been used to predict the potential geographic distribution of many species, including *Liriomyza huidobrensis* (Blanchard), *Bactrocera dorsalis* (Hendel)*, Rhynchophorus ferrugineus* (Olivier) and *Schizaphis graminum* (Rondani)^13,15–17^. No previous study has predicted the potential geographic distribution of *E. fornicates*.

Given that the global climate is changing, we used the CLIMEX model to predict and compare the potential distribution of *E. fornicates* in China with current climate data (1981–2010) and simulated future climate data (2011–2040). We expect our results to help clarify the effect of climate change on the species’ geographic distribution and provide a reference and practical guide to facilitate its control in China.

## Results

### Sensitivity analysis of CLIMEX parameters

**Figure 1:**
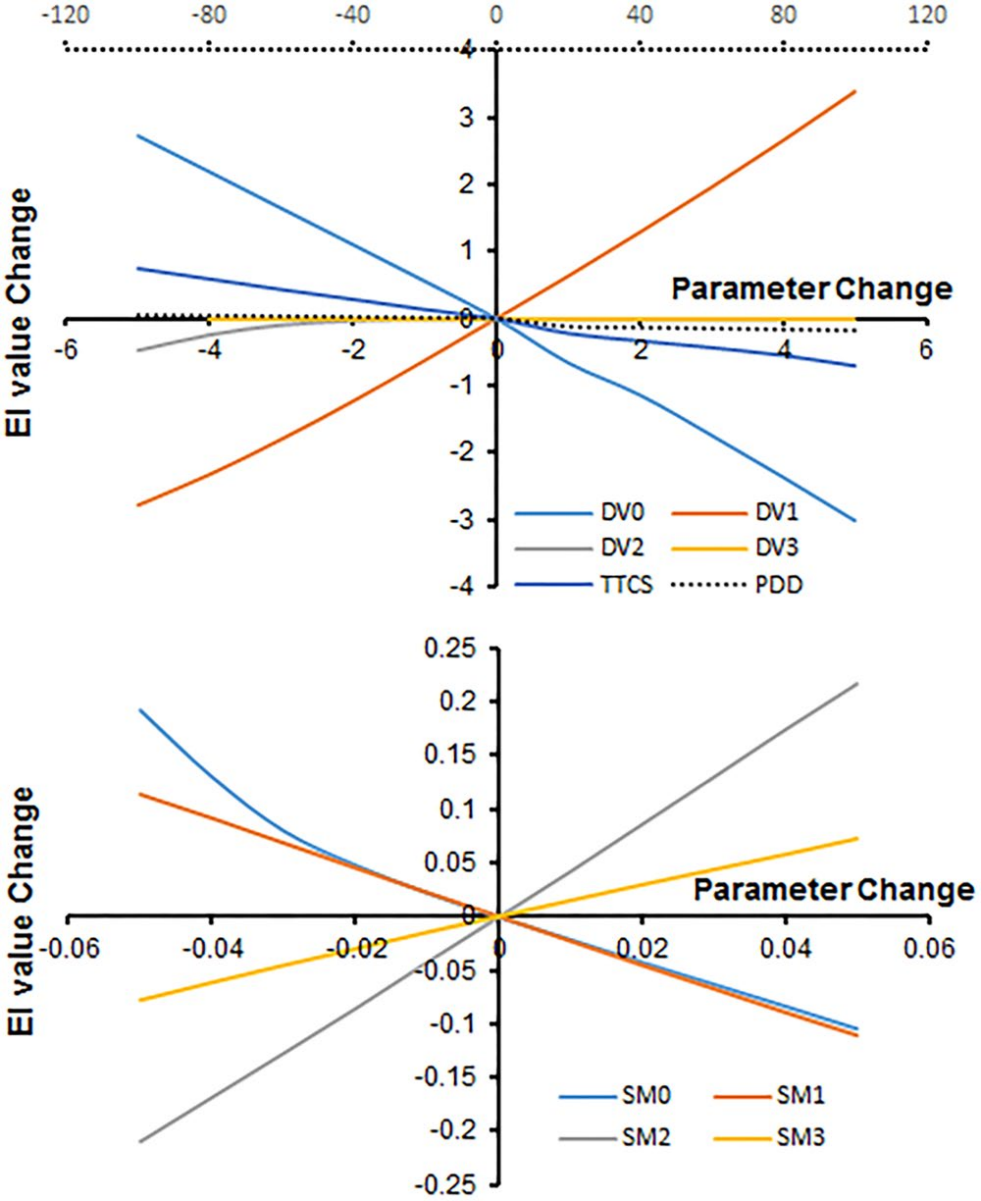
Sensitivity analysis of the selected parameters in CLIMEX for *Euwallacea fornicates* as change in average EI value. The dotted abscissa axis was applied for PDD. (DV0-Lower temperature threshold, DV1-Lower optimum temperature, DV2-Upper optimum temperature, DV3-Upper temperature threshold, TTCS-Cold stress temperature threshold, PDD-Effective accumulated temperature, SM0-Lower soil moisture threshold, SM1-Lower optimal soil moisture, SM2-Upper optimal soil moisture, SM3-Upper soil moisture threshold).

The changes in different parameters from the baseline model and their impact on Eco-climatic Index (EI) values are shown in Fig. 1. Among the six temperature-related parameters, the changes in the parameters DV0 (Lower temperature threshold) and DV1 (Lower optimum temperature) were the most relevant to EI values. The EI value decreases with an increase in the DV0 value, while the EI value increases with an increase in the DV1 value. Changes in other parameters had little influence on EI values. As for the parameters related to moisture, changes to the value of SM0 (Lower soil moisture threshold) and SM1 (Lower optimal soil moisture) had very similar impacts on EI: as the value of these two parameters increased, EI declined. In contrast, EI increased with higher values of SM2 (Upper optimal soil moisture) and SM3 (Upper soil moisture threshold). The results of the sensitivity analysis identified the model parameters that had the most influence on the pest’s distribution.

### Driving variables

**Figure 2:**
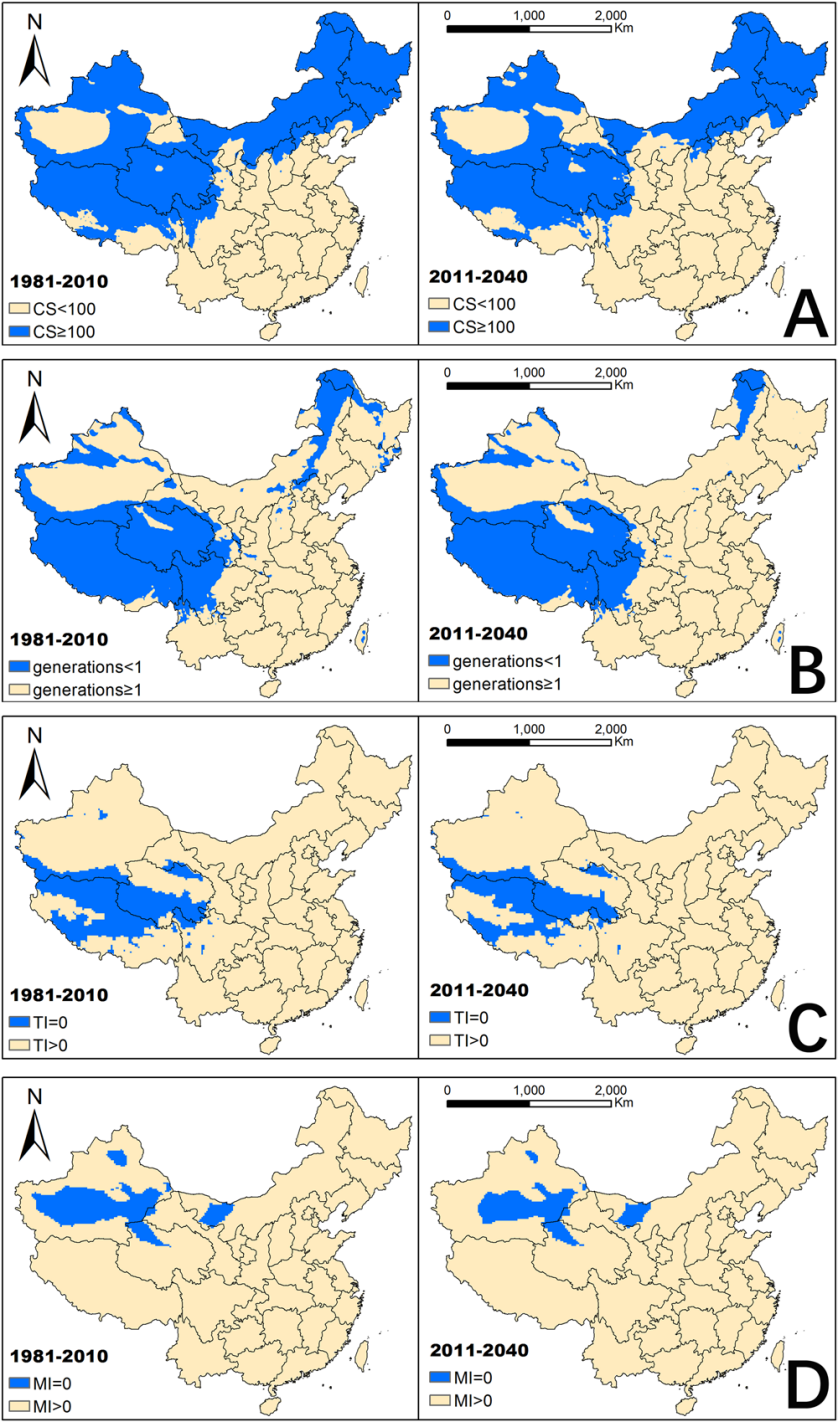
Limiting distribution maps of four different conditions. (**A**) The regions with cold stress (CS) were shown in blue. (**B**) The regions shown in blue couldn’t support for *Euwallacea fornicates* to complete a generation. (**C**). The regions where the Temperature Index (TI) were unsuitable for its survive shown in blue. (**D**) The regions where the Mositure Index (MI) were unsuitable for its survive shown in blue. The related values were exported into GIS software (ArcGIS for Desktop, Software Version 10.2, http://resources.arcgis.com/en/home/) to generate the map in this figure.

**Figure 3:**
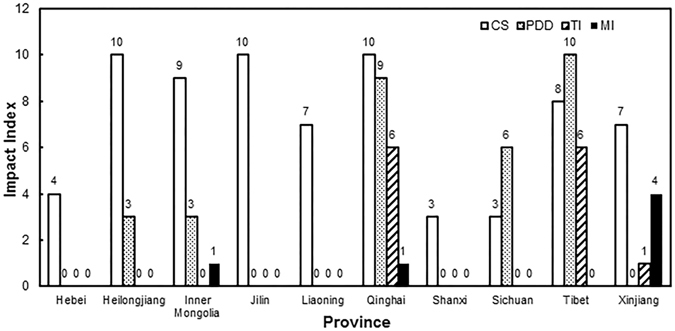
Impact Index in different provinces of the four factors. The range of the index is 0–10. (CS-Cold stress, PDD-Effective accumulated temperature, TI-Temperature Index, MI-Mositure Index).

We calculated the EIs for *E. fornicates* using the CLIMEX model under current and future climate conditions. The EI value will be equal to 0 when the output index satisfies one of the following four conditions: (1) Generations < 1 (calculated by PDD); (2) Cold Stress (CS) ≥ 100; (3) Temperature Index (TI) = 0; and (4) Moisture Index (MI) = 0. The regions that satisfy the four conditions are shown in Fig. 2. The impact of each condition in each province was measured in the range 0–10 (Impacted Index) and the results are shown in Fig. 3 (under current climate conditions), according to the percentage of the area influenced by the condition. It is obvious that CS and PDD (Effective accumulated temperature) are the main limiting factors of current and future distribution of *E. fornicates* in China. Among the four conditions, CS is the most important factor limiting the distribution in northeast China and Inner Mongolia. Both PDD and CS are limiting factors in Tibet, Qinghai, most regions of Gansu, and northwest Sichuan, while TI is the limiting factor for distribution in Tibet and Qinghai, and MI is the limiting factor for central Xinjiang and western Inner Mongolia.

### Potential distribution under current climate (1981–2010)

**Figure 4:**
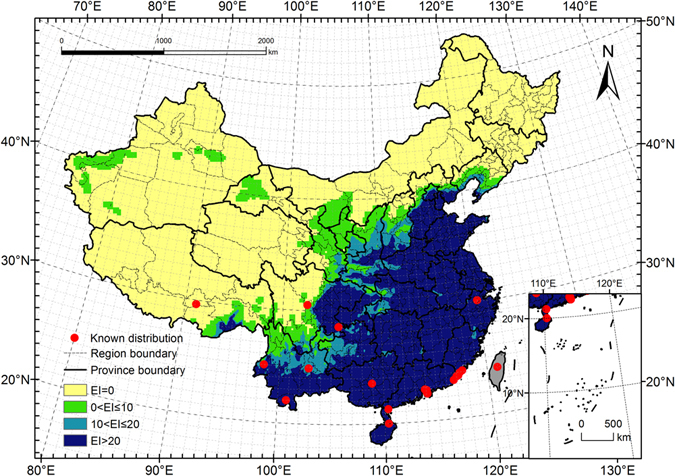
Potential distribution for *Euwallacea fornicates* under the current climate (1981–2010). Yellow regions indicated unsuitable habitats (EI = 0), green regions indicated marginally favorable habitats (0 < EI ≤ 10), turkish blue regions indicated favorable habitats (10 < EI ≤ 20) and deep blue regions indicated highly favorable habitats (EI > 20). The red points showed the locations of the known distribution in China. The grey regions (Taiwan) were not analyzed. The CLIMEX results were exported into GIS software (ArcGIS for Desktop, Software Version 10.2, http://resources.arcgis.com/en/home/) to generate the map in this figure.

The Ecoclimatic Index (EI) values for each grid point were calculated using CLIMEX and current climate data. These values were then imported to ArcMap to map the potential distribution of *E. fornicates* in China (Fig. 4). After reclassifying for different climatic favourability levels, we estimated the potential distribution of *E. fornicates* to include 3.76 million km^2^ primarily located in southern and northern China. This represents 39.1% of the total area of mainland China.

#### Highly favourable habitat

The total area of highly favourable habitat amounted to 2.55 million km^2^, which is 26.5% of the total mainland area and 67.8% of the total potential distribution. This area includes the provinces of Hainan, Guangdong, Guangxi, Shandong, Jiangsu, Jiangxi, Shanghai, Hongkong and Tianjing as well as large areas of Anhui, Fujian, Hunan, Zhejiang, Henan, Hubei, Chongqing and Guizhou. Additional favourable habitat was identified south of Beijing, Yunnan, Shanxi and Shaanxi, south and east of Hebei, and east of Sichuan. In addition, there are scattered pockets of predicted habitat in parts of Liaoning, Gansu and Tibet.

#### Favourable habitat

The area of favourable habitat amounted to 0.50 million km^2^, or 5.2% of the total mainland area and 13.2% of the total potential distribution. This includes most of Shaanxi, the northern part of Yunnan, the western region of Guizhou and Hubei, and the southern and north-eastern parts of Sichuan. Additional areas fall in eastern Chongqing, south-eastern Gansu, southern Shanxi, Henan, and Liaoning, and parts of Tibet, Hebei, Beijing, and other provinces in southern and northern China.

#### Marginally favourable habitat

The area of marginal habitat amounted to 0.71 million km^2^, or 7.4% of the total mainland area and 19.0% of the total potential distribution. The area includes northern Yunnan and Beijing, southern Sichuan, northern Shaanxi, most of Ningxia and parts of Guizhou, Hubei, Chongqing, Gansu, Shanxi, Hebei and Liaoning. Additional pockets fall southeast of Tibet, Xinjiang, Inner Mongolia and Heilongjiang.

### Potential distribution under future climate conditions (2011–2040)

**Figure 5:**
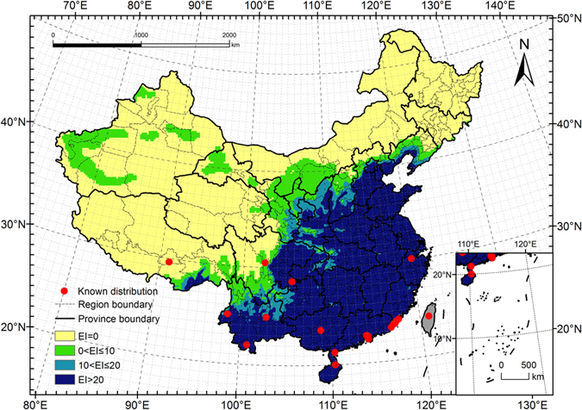
Potential distribution for *Euwallacea fornicates* under the future climate (2011–2040). The future climate data were derived from CSIRO-Mk3-6-0 models (RCP8.5). Yellow regions indicatef unsuitable habitats (EI = 0), green regions indicated marginally favorable habitats (0 < EI ≤ 10), turkish blue regions indicated favorable habitats (10 < EI ≤ 20) and deep blue regions indicate highly favorable habitats (EI > 20). The red points showed the locations of the known distribution in China. The grey regions (Taiwan) were not analyzed. The CLIMEX results were exported into GIS software (ArcGIS for Desktop, Software Version 10.2, http://resources.arcgis.com/en/home/) to generate the map in this figure.

**Figure 6:**
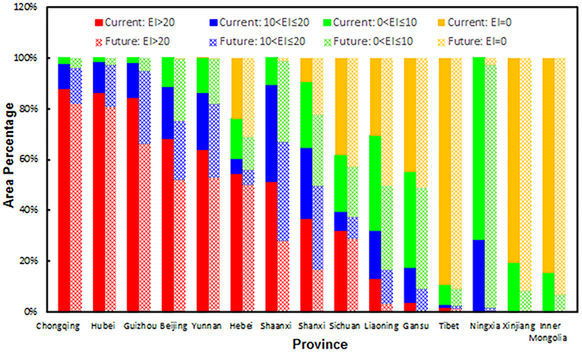
Area changes of different ranges of EI value in different provinces for *Euwallacea fornicates* under the current and future conditions. Almost all the area of Anhui, Fujian, Guangdong, Guangxi, Hainan, Henan, Hongkong, Hunan, Jiangsu, Jiangxi, Shandong, Shanghai, Tianjin and Zhejiang are highly favourable habitats, and all the area of Jilin, Qinghai and Heilongjiang are unfavourable habitats, therefore, these provinces weren’t illustrated in the figure.

Under future climate conditions, the total potential distribution of *E. fornicates* was projected to cover approximately 4.16 million km^2^, or 43.4% of the total mainland area. The overall distribution is similar to that predicted for current climate conditions (Figs 4 and 5), and the distributions of favourable areas within some provinces showed considerable differences (Fig. 6). The areas of highly favourable habitats in many provinces may decrease to different extents, especially in Guizhou, Beijing, Shaanxi, and Shanxi. In addition, the total potential distribution in almost all provinces was projected to show a decreasing trend. It is obvious that the climate in each province may become less suitable for pest survival in the future.

### Comparison of distributions under current and future climate conditions

#### Area of potential distribution

**Figure 7:**
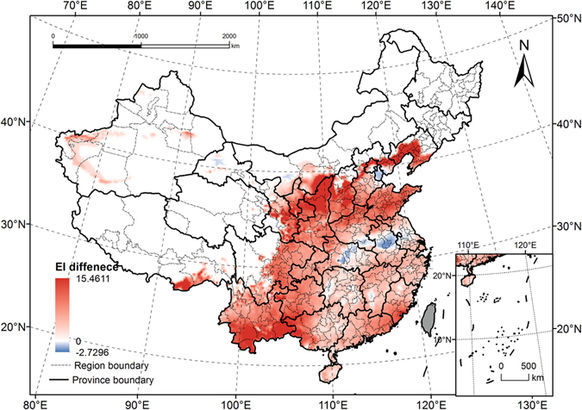
Change of the EI values under current (1981–2010) and future (2011–2040) conditions in China. Red means increase, blue means decrease, the deep of the color show the change degree of EI values. The CLIMEX results were exported into GIS software (ArcGIS for Desktop, Software Version 10.2, http://resources.arcgis.com/en/home/) to generate the map in this figure.

There were minor differences in the distribution of favourable habitats with different levels of climate favourability (Fig. 7). Under future climate conditions, the total potential distribution was predicted to increase by 0.41 million km^2^. Specifically, the areas of highly favourable and marginal habitats were predicted to increase by 0.25 million km^2^ and 0.11 million km^2^, while the area of favourable habitat may decrease by 0.05 million km^2^. Thus, the main change predicted is an increase in the area of highly favourable habitat.

#### Range shifts

When predicted distribution under current and future climate scenarios was compared, the highly favourable habitat was projected to tend to shift north about 0.3–2° latitude in Sichuan, Shanxi, Shaanxi and Liaoning. By contrast, the range may shift west in Guizhou. Favourable habitat spread to the north about 0–1.3°, especially in Shaanxi. Marginally favourable habitat shifted north 0.5–4.8° in Inner Mongolia, Shanxi, Hebei and Liaoning. There may be also small changes in the provinces of western China.

#### Changes in Climate favourability

**Figure 8:**
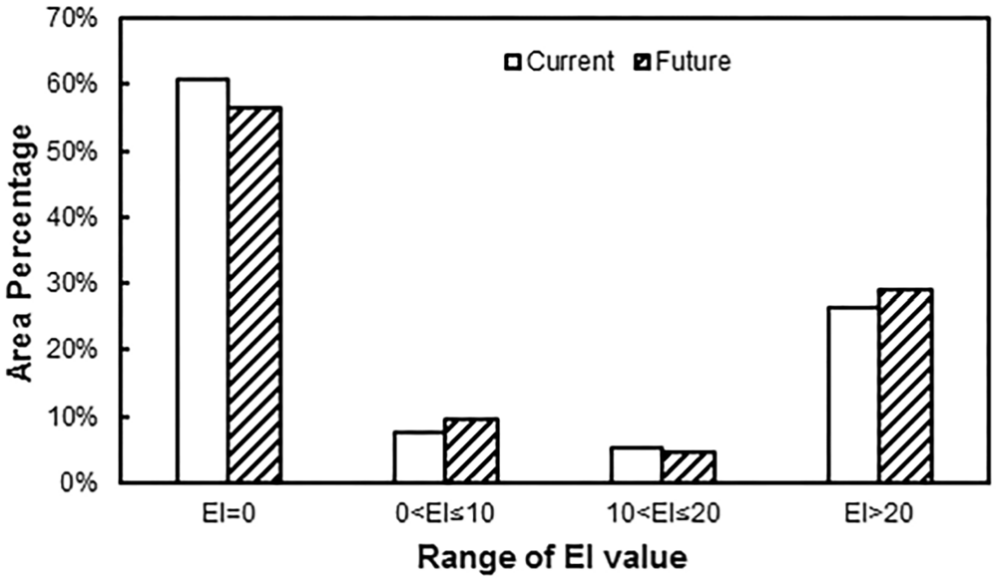
Area proportion of different ranges of EI values for *Euwallacea fornicates* under current and future climate conditions.

We calculated the difference between the EI values under both climate scenarios and interpolated to map the change in habitat favourability (Fig. 8). The different colours represent the difference in EI values (red means increase, blue means decrease). Under future climate conditions, the favourability over most of *E. fornicates*’ potential distribution was projected to increase. Only in scattered regions of western Gansu may favourability likely decrease slightly (change in EI < 3).

The regions with the largest predicted increase in EI values are mainly located in southern Yunnan, south-eastern Gansu, northern Shaanxi, and southern Liaoning. EI was predicted to increase as high as 15.46. In other regions, EI values may increase by 2~5 units on average.

## Discussion

**Figure 9:**
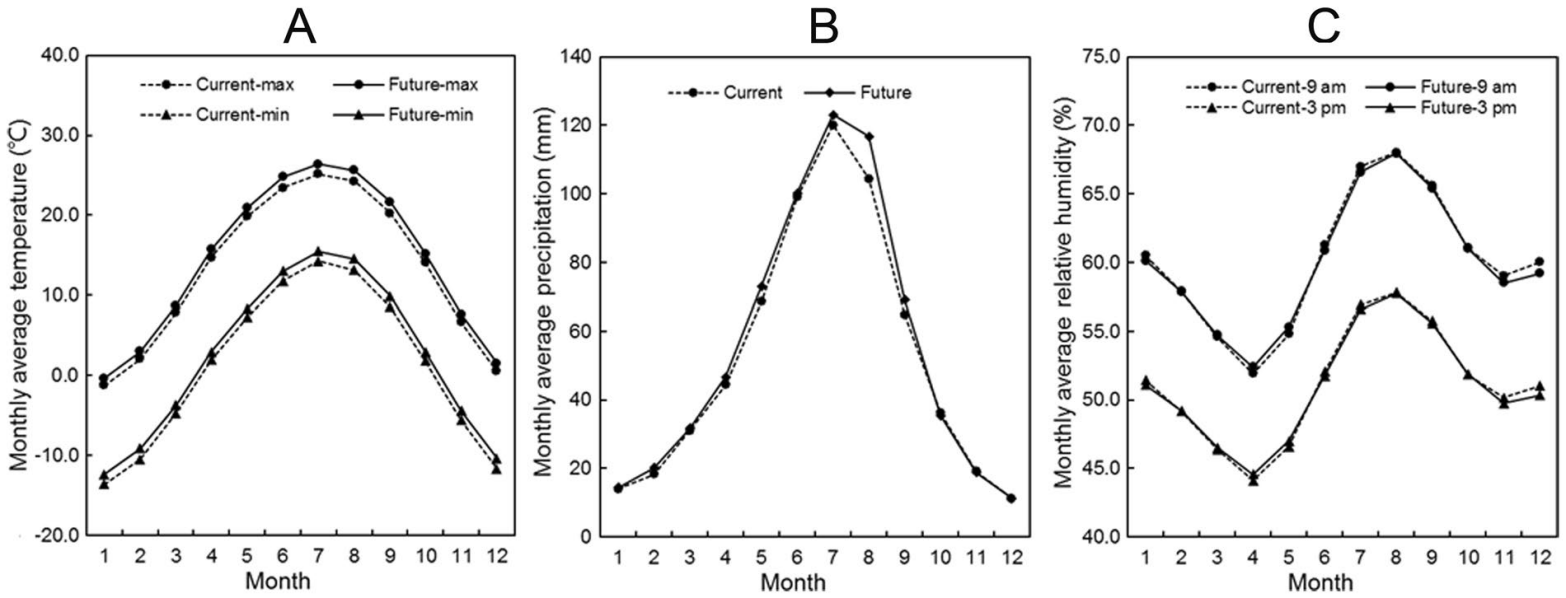
Monthly average climate data under the current (1981–2010) and future (2011–2040) conditions in China. The five groups of climate data which were divided into three classes were the current and future climate data we used to predict the potential distribution in this study.

Climate is one of the most important factors limiting species distribution. With global warming, the climate in China is also predicted to change correspondingly. Current and future climate data (temperature, precipitation, and relative humidity) calculated in this study are compared in Fig. 9. The results show that the monthly average maximum and minimum temperatures are projected to increase in the period 2011–2040; the maximum temperature may increase by about 1 °C while the minimum temperature may increase by about 1.2 °C. The increase in temperature in the summer (July to September) may be the largest. Monthly average precipitation may increase from January to September. It is unclear whether monthly average relative humidity will change.

**Figure 10:**
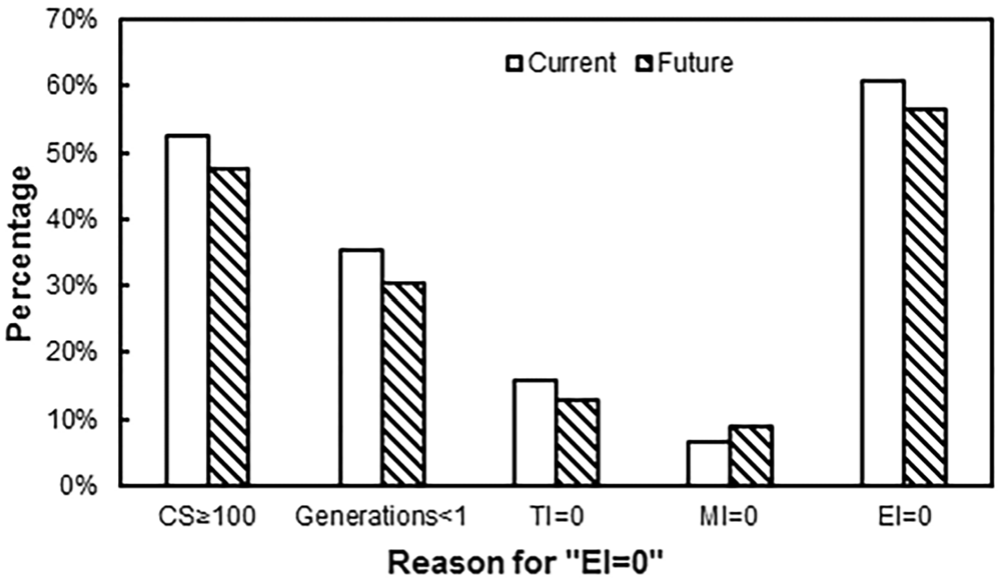
Percentages of four different reasons for “EI = 0” under current and future climate. “Percentage” represents for the proportion of grid points which satisfy the condition to cause “EI = 0”. “generation < 1” means the climate data in this region couldn’t meet the development of a generation. “CS ≥ 100” means the species couldn’t survival in this region for cold stress. “TI” means Temperature Index, “MI” means Mositure Index. While “TI = 0” or “MI = 0” will cause the “GI = 0” (Growth Index) and lead to “EI = 0”.

To analyse the impact of climate change on *E. fornicates*, we compared the proportion of grid points under the four conditions, which resulted in “EI = 0” (Generations < 1, CS ≥ 100, TI = 0 and MI = 0) under current and future climate conditions (Fig. 10). As a whole, the percentage of grid cells (percentage of total area) with “EI = 0” will decrease, while the percentage under all four conditions will also decrease. As for generation, the increase in temperature will increase the accumulated temperature in a region, resulting in increases in the generation length; thus, the number of regions where the accumulated temperature can no longer support the completion of the development of one generation (generations < 1) will decrease. Temperature increases in the winter will reduce CS, and the number of regions that are unsuitable as habitats because of cold stress will decrease. In addition, increases in temperature and precipitation will increase the Temperature Index and Moisture Index in the regions and lead to fewer regions with TI or MI equal to zero. These results also show that temperature is the most important meteorological factor affecting insect distribution.

Non-climatic factors, which are not included in CLIMEX, can also affect species distribution, such as natural enemies, topography, and host distribution. Among them, host distribution is one of the most important factors influencing a pest’s potential distribution, because hosts are a necessary condition for pest survival. However, the host trees of *E. fornicates* are diverse and include *Heavea brasiliensis*, *Ricinus communis*, *Robinia pseudoacacia*, *Cassia siamea*, *Platanus acerifolia*, *Acer negundo*, *Acacia* sp., *Cinnamomum* spp., *Populus* spp., *Quercus* spp., and *Salix* spp., Over 100 species^21^ are found in China. Most of these plants are mainly located in southern China, while *Populus* spp. and *Salix* spp. are widely distributed. Nevertheless, *E. fornicates* is very resilient and may change its feeding preferences with changes in climate. Thus, its distribution may not be limited by the distribution of its hosts.

**Figure 11:**
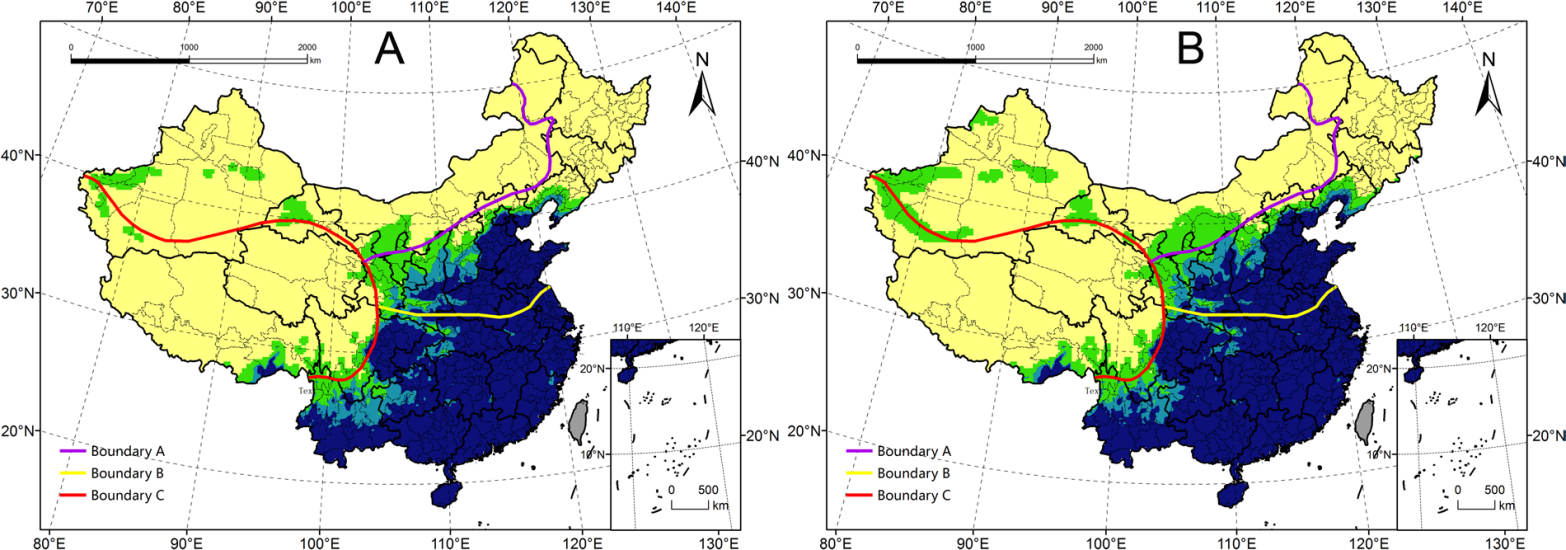
Potential distribution maps with geographic boundary lines. Purple line (Boundary **A**) is the boundary of the Region and North Region, yellow line (Boundary **B**) is the boundary of the South Region and North Region, red line (Boundary **C**) is the boundary distinguishing the Qinghai-Tibet Region with other three regions. The maps in this figure were generated by GIS software (ArcGIS for Desktop, Software Version 10.2, http://resources.arcgis.com/en/home/) with map layer overlapping.

Based on geographic differences in environmental and socioeconomic characteristics, China could be divided into four areas: South Region, North Region, Northwest Region and Qinghai-Tibet Region (Fig. 11). The Daxing’an-Yinshan-Helan Mountains (boundary A) divided north and northwest regions approximately by the 400 mm isohyet. The Qinling-Huaihe boundary line (boundary B) is the boundary of the South Region and North Region, mainly determined by climate, which is overlap with isothermal line of 0 °C in January and 800 mm isohyet. The Kunlun-Qilian-Hengduan Mountains (boundary C) form the boundary that separates the Qinghai-Tibet Region from the other three regions, mainly divided by terrain. Most of the determined factors which divided the four regions are the main factors which limit insects’ distribution. Looking at the potential distribution of *E. fornicates* (Fig. 11), we found that it should mainly be distributed in areas near boundaries A and C (except for Jilin and Heilongjiang). Considering the determined factor of the two boundaries, terrain and rainfall may be the main factors which limit the distribution *E. fornicates*. As for terrain, the elevation on either side of boundary C is markedly different. Elevation is an important factor because it influences temperature. The Qinghai-Tibet Region’s elevation is much higher than other regions, and so its average temperature is much lower. There, the annual accumulated temperature does not permit the development of one generation, making most of the region unsuitable for *E. fornicates*. Indeed, boundary C may be a boundary to the potential distribution for many kinds of insects for this reason. These two boundaries (A and C) could be treated as a reference when projected the potential distribution.

However, awareness of *E. fornicates* is low in China and there is little research on this pest. Therefore, we provide two suggestions to reduce the likelihood of *E. fornicate* spreading. First, we should strengthen surveillance and monitor its potential distribution. Surveillance for *E. fornicates* should be conducted in every region of China, and its potential distribution should be monitored over the long term. Second, we should strengthen quarantine protocols, especially where goods are imported from areas of *E. fornicates* outbreaks. This pest spreads both through dispersal of adults and transport of its hosts. Imported fruits, host plants and wooden packaging that come from America and Vietnam should be strictly checked.

## Methods

### CLIMEX model

CLIMEX is a dynamic simulation model that estimates the potential geographical distribution and relative abundance of a species according to climate^19^. It is widely used for species of plants and insects^15–18,20–22^. The Compare Locations function of CLIMEX requires physiological data and known distribution data to determine the CLIMEX parameters needed for a species to survive. After climate data are imported into CLIMEX, the potential distribution of the species can be predicted with the CLIMEX parameters. This study used the Compare Locations function in CLIMEX 4 to simulate the results^19^.

The CLIMEX parameters can be divided into three classes. The three classes determine the Annual Growth Index (GI_A_), the Annual Stress Index (SI) and limiting factors, such as sufficient degree-days (DD) to complete the lifecycle and diapause. These are integrated into an Ecoclimatic Index (EI) that describes the favourability of a location for a species. The GI_A_ describes the potential for population growth. The SI limits survival during the unfavourable season and determines the boundary of a species’ geographical distribution^19^.

1 $${\rm{EI}}=G{I}_{A}\times SI\times SX$$

2 $${\rm{SI}}=(1-\frac{{\rm{CS}}}{100})(1-\frac{{\rm{DS}}}{100})(1-\frac{{\rm{HS}}}{100})(1-\frac{{\rm{WS}}}{100})$$

where, in equation (1), GI_A_ is mainly determined by Temperature Index (TI), Moisture Index (MI) and Diapause Index (DI) (only for diapause species). SX, the Stress Interaction Index, is usually not considered in practice. In equation (2), CS, DS, HS, and WS represent annual cold, dry, heat and wet stress indices, respectively^19^.

The EI is scaled between 0 and 100, with an EI close to 0 indicating that the location is not favourable for the long-term survival of the species. EI values of 100 are only achievable under constant and ideal conditions, such as in incubators.

#### ArcMap software

ArcMap is one of three desktop components of ArcGIS (Environment System Research Institute, ESRI) Desktop. ArcMap is professional mapping software with all the functions for map making, map editing, map analysis, etc^23^. This study used the functions of inverse distance-weighted (IDW) interpolation, reclassification and thematic mapping to visualise the results.

### Data collection

#### Known distribution of Euwallacea fornicatus

**Figure 12:**
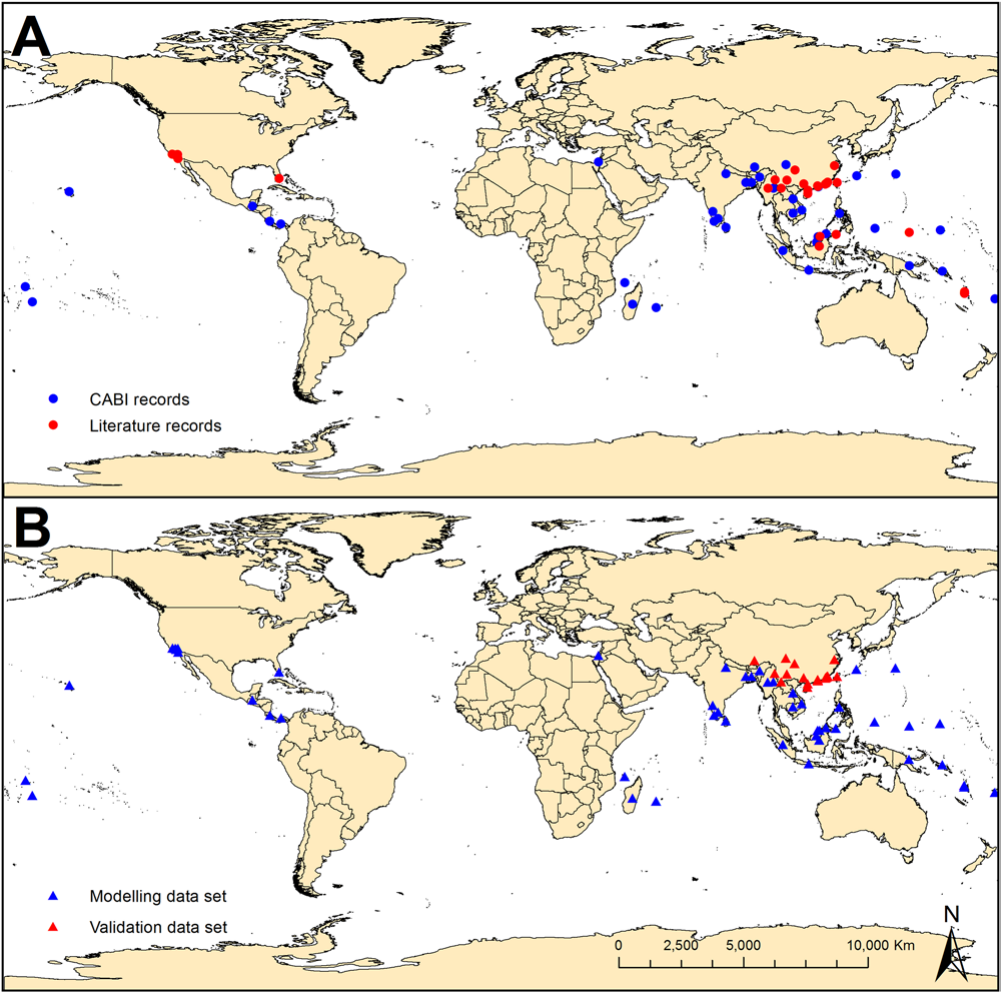
Global known distribution of *Euwallacea fornicates*. (**A**) Showed the data sources. The red dots were the locations recorded in references, the blue dots were the locations recorded in CABI. (**B**) Showed the usefulness of data. The red triangle showed the locations of validation data set, the blue triangle showed the locations of modeling data set. The map in this figure was generated by GIS software (ArcGIS for Desktop, Software Version 10.2, http://resources.arcgis.com/en/home/).

The known distribution data were mainly ascertained from the Commonwealth Agricultural Bureaux International (CABI) distribution map^1^ (Fig. 12A, shown in blue), with additional data from the literature (Fig. 12A, shown in red). A known distribution map of *E. fornicates* was made with 73 point locations obtained from CABI website and literatures (Fig. 12A, shown in red and blue), distributed across Asia, Africa, North America, Oceania, Central America and the Caribbean, including 28 countries. This also included detailed distribution data for 11 provinces of China, such as Guangxi, Guangdong, Yunnan and Fujian provinces (see Supplementary Table S1). Both native and exotic records of existence were collected and treated as the total known distribution of the pest. In order to verify the validity of the model, we selected the current known distribution in China (20 points) (Fig. 12B, shown in red) from all the distribution data as a validation data set, and other data were used for model fitting (Fig. 12B, shown in blue).

#### Current climate data

The CLIMEX database contains data from 2031 global meteorological stations (only 85 stations are located in China) and gridded high-resolution datasets (0.5° and 1°). The data include average maximum monthly temperatures, average minimum monthly temperatures, and average monthly precipitation and relative humidity at 09:00 h and 15:00 h. The time periods of the datasets are 1961–1990 and 1950–2000, respectively^17,19^.

Using appropriate climate data is important for the accuracy of the predictions. When predicting the current potential distribution of a species, most Chinese scholars use meteorological data (1971–2000, 1981–2010 or 1951–2000) published on China’s Meteorological Data website^24–27^. By contrast, most foreign scholars choose grid climate data (1950–2000) after interpolation, which is included in CLIMEX4^17,28,29^. The China Surface Climate Monthly Standard Values dataset (1981–2010) (without Taiwan), published on the China Meteorological Data website (http://data.cma.cn/), includes data from 1866 meteorological stations. We chose this dataset because it includes more meteorological stations and more recent climate data.

Five groups of data were extracted from all the observed meteorological data in the dataset, and then high-resolution data were obtained using ANUSPLIN software^30^ to interpolate the climate data over elevation, thus allowing the effect of elevation on temperature to be taken into consideration. This avoided errors caused by nonuniform climate data. The gridded data were interpolated to a spatial resolution of 8 km × 8 km, resulting in a total of 148,203 grid cells across China^31^. The interpolation accuracy was assessed and showed high accuracy (P-value < 0.001). The results of the assessments of annual average climate data (five groups) can be found in Supplementary Fig. S1, while the results of the entire assessments of monthly average climate data (60 groups) are shown in Supplementary Table S2.

#### Future climate data

The future climate data in CLIMEX were based on the GCMs from the Coupled Model Intercomparison Project phase 3 (CMIP3) and the emissions scenarios in the IPCC’s Special Report Emissions Scenarios (SRES)^29^. Although there was not much difference between future climate data simulated by CMIP3 and CMIP5, the global climate models (GCMs) in CMIP5 add a carbon cycle module that models the relationship between carbon dioxide emissions and increases in temperature^32^. Thus, in specific regions, there is a difference between the predictions of CMIP3 and those of CMIP5. We chose to use simulated climate data from CMIP5 as the newest simulation to predict the potential distribution of *E. fornicates* under future climate conditions.

CMIP5 contains 46 GCMs projected by different countries using different methods. It provides forecasts of climate in the 21^st^ century and beyond. The Representative Concentration Pathways (RCPs) is a new set of scenarios, a new set of scenarios, which was used for the new climate model simulations carried out under the framework of CMIP5, including RCP2.6, RCP4.5, RCP6.0 and RCP8.5. Among these, RCP8.5 is the most pessimistic greenhouse gas emissions scenario and predicts the greatest rises in temperature^14^.

Predictions of long-term climate change are highly variable, so the next 30 years (2011–2040) were focused upon. When selecting GCMs, we focused on the accuracy of temperature, precipitation and relative humidity that different models simulated for the historical climate in China. As for temperature, many studies have reported that almost all of the models result in highly accurate temperature simulations in China. However, relative humidity prediction is not so accurate^33^. The accuracy of precipitation prediction is a critical factor when we select an appropriate model. In China, the CSIRO model provides a model that can accurately simulate precipitation^34^. We outputted simulations of the five data sets (average minimum monthly temperatures, average monthly precipitation, average maximum monthly temperatures, and relative humidity at 09:00 h and 15:00 h) for China using almost all of the models in CMIP5 (41 models). To compare the results to the observation data, the CSIRO-Mk3-6-0 (Commonwealth Scientific and Industrial Research Organization, Australia) was finally chosen, with a resolution of 192 × 96. The RCP8.5 scenario was chosen to assess the change of the species’ potential distribution under significant climate warming.

With the selected model, scenarios and simulated time period, the appropriate climate data from CMIP5 were extracted. This was computed as the difference between the output of the GCM run for the baseline years (1981–2005) and for the target years (2011–2040). These changes were interpolated to a grid with a high (8 km) resolution with ANUSPLIN software. The next step was “calibration”, which is a necessary step because GCMs do not accurately predict the current climate in all places. For this reason, observed current climate could not be directly compared with predicted future climate. It is also problematic to compare the response to simulated current conditions with a response to simulated future conditions because the simulated current conditions could be far from reality. Therefore, current climate data (1981–2010) were treated as baseline data, and then the interpolated climate change data were added, to obtain calibrated future climate data^35^.

#### Physiological data

In India, Kumar *et al*.^36^ observed the physiological characteristics of *E. fornicates* and found that the pest was most active when the prevailing temperatures were between 26 and 35 °C and the relative humidity was 75–95%.

In Australia, Walgama and Zalucki^37^ reared individuals under a range of constant temperatures (15–32 °C) to study the effect of temperature on the rate of development of *E. fornicates*. Estimates of lower development thresholds were obtained for eggs (15.7 ± 0.5 °C), larvae (15.8 ± 0.8 °C) and pupae (14.3 ± 1.4 °C). DD for development were 70 ± 4.4, 95 ± 8.5 and 72 ± 5.1, respectively. About 237 DD were required for development from egg to adult emergence, and ~136 DD were needed for the preoviposition period, for a total of 373 DD. This could be regarded as the amount of heat required for the development of one generation.

The lower development thresholds estimated by Danthanarayana^38^ were 15, 16 and 14 °C for eggs, larvae and pupae, respectively. The thermal constants for eggs, larvae and pupae were 67, 100 and 72, respectively.

Gadd^39^ indicated that immature stages cannot survive at temperatures below 15 °C. All these observations suggest that the optimum temperature range for both eggs and pupae is 28–30 °C.

### Research method

#### Overall analysis process

To study the potential distribution of *E. fornicates*, there were three main steps.

The first step is to set the CLIMEX parameters. The climate type of the original habitat (Southeast Asia) of *E. fornicates* is tropical monsoon climate and tropical rainforest climate, so, when a new species was created in CLIMEX, the wet tropical template was used as a reference. The CLIMEX parameters were then fitted based on the physiological data and the modelling data set. The parameters required for *E. fornicates* survival (Table 1) were defined when the result predicted by CLIMEX was consistent with the actual known distribution.

**Table 1:** CLIMEX parameter values for *Euwallacea fornicates*. The values in Wet Tropical Template came from CLIMEX, and the values in the middle column were from the literature.

CLIMEX parameter	Wet tropical template	Literature	Final parameter
DV0-Lower temperature threshold	15	12.9–16.2	15
DV1-Lower optimum temperature	28	26	26
DV2-Upper optimum temperature	33	35	35
DV3-Upper temperature threshold	36	—	40
SM0-Lower soil moisture threshold	0.35	—	0.05
SM1-Lower optimal soil moisture	0.7	—	0.3
SM2-Upper optimal soil moisture	1.5	—	1
SM3-Upper soil moisture threshold	2.5	—	2.5
TTCS-Cold stress temperature threshold	2	—	−10
THCS-Cold stress temperature rate	0	—	−0.005
TTHS-Heat stress temperature threshold	37	—	42
THHS-Heat stress temperature rate	0.0002	—	0.0002
SMDS-Dry stress threshold	0.25	—	0.05
HDS-Dry stress rate	0.01	—	−0.001
SMWS-Wet stress threshold	2.5	—	2.5
HWS-Wet stress rate	0.002	—	0.005
PDD-Effective accumulated temperature	0	355–391	373

The second step was to calculate EI values in China under current and future climate. After the appropriate CLIMEX parameters were set, the current and future climate data were imported into CLIMEX and then the “compare locations (1 species)” function was chosen to output the EI values of each grid point for the two climate scenarios.

The final step consisted of drawing and analysing maps using ArcMap. The EI was then imported into ArcMap, and the IDW and thematic mapping functions were used to draw the potential distribution maps of *E. fornicates* under current and future climate. The distributions were then calculated and analysed using ArcMap.

#### Fitting CLIMEX parameters and sensitivity analysis

When setting parameters, the SI was first debugged to define the borders of the potential distribution and to define the specific distribution based on the GI_A_ and on limiting conditions.

Cold Stress/Heat Stress (CS/HS). In CLIMEX, the values of CS/HS will influence the northern and southern borders of the potential distribution of a species. CS is defined by the Cold Stress Temperature Threshold (TTCS) and the Cold Stress Temperature Rate (THCS). No previous studies have evaluated the cold tolerance of *E. fornicates*, so parameter values of species of the Scolyiidae (*Dendroctonus valens*, *D. frontalis*, *Trypodendron domesticum*) were adopted for reference^40–42^. After debugging, TTCS and THCS were set to −10 °C and −0.005 week^−1^. Because *E. fornicates* is a tropical species, it can tolerate high temperatures; therefore, the Heat Stress Temperature Threshold (TTHS) and Heat Stress Temperature Rate (THHS) were set to 42 °C and 0.0002 week^−1^.

Dry Stress/Wet Stress (DS/WS). According to previous reports, *E. fornicates* prefer to live in environments with high relative humidity. Therefore, the wet tropical template was used as a reference, and the Wet Stress Threshold (SMWS) and Wet Stress Rate (HWS) were set to 2.5 and 0.005 week^−1^. However, *E. fornicates* is highly adaptable, surviving and spreading in California^4^, where annual average precipitation and relative humidity are low. For this reason, the Dry Stress Threshold (SMDS) and Dry Stress Rate (HDS), which determined the DI values, were set to 0.05 and −0.001 week^−1^.

GI_A_ was defined by the Temperature Index (TI) and Soil Moisture Index (SMI). The four parameters that defined the TI are Lower Temperature Threshold (DV0), Lower Optimum Temperature (DV1), Upper Optimum Temperature (DV2) and Upper Temperature Threshold (DV3). These were parameterised based on physiological data in literature. The literature reported that immature stages cannot survive at temperatures below 15 °C and the lower development thresholds for each stage are around 15 °C; thus DV0 was set to 15 °C. Field observations found that the optimum temperature range for *E. fornicates* is between 26 and 35 °C, so DV1 and DV2 were set to 26 °C and 35 °C, while DV3 was set to 40 °C. The four parameters that defined the SMI were Lower Soil Moisture Threshold (SM0), Lower Optimal Soil Moisture (SM1), Upper Optimal Soil Moisture (SM2) and Upper Soil Moisture Threshold (SM3). Based on SMWS and SMDW, these four parameters were set to 0.05, 0.3, 1 and 2.5 after debugging.

Based on Walgama and Zalucki^37^ and Danthanarayana^38^, the amount of heat required for development of one generation ranged between 355 and 391 DD; thus the effective accumulated temperature was set to 373 DD.

CLIMEX parameter values are listed in Table 1. We also undertook an analysis of the sensitivity of parameters to different values (see Supplementary Table S3), following the approach of Vanhanen *et al*.^44^ and Taylor and Kumar^43^.

#### Classification of EI values

In practice, EI values are classified to describe the favourability of a region for a species in more detail. The standard of classification is species-dependent and should be defined in accordance with actual occurrence in different regions. However, with little known distribution data for *E. fornicates*, it is hard to classify the EI values. Sutherst^45^ suggested that EI < 10 indicates that a location is marginal for a species and that values in excess of 20 support substantial population densities in practice. On this basis, the EI values were grouped into four arbitrary classes: unfavourable (EI = 0), marginally favourable (0 < EI ≤ 10), favourable (10 < EI ≤ 20) and highly favourable (EI ≥ 20) habitats.

#### Model validation

Among the various methods used for model validation, visual validation (the iterative geographic fitting procedure) is the one suggested by creators of the model and it has been used in many studies^46–49^. In this study, we used the modelling data set to fit the parameters and then used the validation data set to compare the potential distribution and current known distribution with respect to geographical range. As shown in Fig. 3, 95% of the occurrence records fall within suitable categories, with only one record in Tibet predicted to be an unsuitable habit. A comparison of the results confirmed that the selected CLIMEX parameters for the pest were optimum.

## Electronic supplementary material

Supplementary Information
