# Author Correction: Predicting the potential distribution in China of *Euwallacea fornicatus* (Eichhoff) under current and future climate conditions

**DOI:** 10.1038/s41598-018-23580-3

**Published:** 2018-04-05

**Authors:** Xuezhen Ge, Chao Jiang, Linghong Chen, Shuang Qiu, Yuxiang Zhao, Tao Wang, Shixiang Zong

**Affiliations:** 1 Key Laboratory of Beijing for the Control of Forest Pests, Beijing Forestry University, Beijing, 10083 P.R. China; 2 College of Forest Science, Beijing Forestry University, Beijing, 100083 P.R. China; 3 College of Environment and Resources, Jilin University, Changchun, 130012 P.R. China; 4 Department of Afforestation, National Forestry Bureau, Beijing, 100714 P.R. China; 5 Mentougou Forestry Station, Beijing, 102300 P.R. China

Correction to: *Scientific Reports* 10.1038/s41598-017-01014-w, published online 19 April 2017

The original version of this Article contained a typographical error in the Title, where “*Euwallacea fornicatus*” was incorrectly given as “*Euwallacea fornicates*”.

This has now been corrected in the HTML and PDF versions of this Article.

