# Supplementary material for: Predicting the potential distribution in China of Euwallacea fornicatus (Eichhoff) under current and future climate conditions
Source: Sci Rep. 2017 Apr 19;7:906. doi: 10.1038/s41598-017-01014-w (PMC5430415; doi:10.1038/s41598-017-01014-w)
Supplement: Supplementary file 1 — Supplementary Information [file 41598_2017_1014_MOESM1_ESM.pdf]

Supplementary Information

Predicting the potential distribution in China of *Euwallacea fornicatus* (Eichhoff) under current and future climate conditions

Xuezhen Ge[1], Chao Jiang[2], Linghong Chen[3], Shuang Qiu[4], Yuxiang Zhao[4], Tao Wang[5], Shixiang Zong [1]*

[1]Key Laboratory of Beijing for the Control of Forest Pests, Beijing Forestry University, Beijing 10083, P.R. of China

[2]College of Forest Science, Beijing Forestry University, Beijing 100083, P.R. of China

[3]College of Environment and Resources, Jilin University, Changchun 130012, P.R. of China

[4]Department of Afforestation, National Forestry Bureau, Beijing 100714, P.R. of China

[5] Mentougou Forestry Station, Beijing 102300, P.R. of China

*Corresponding authors: Shixiang Zong, E-mail: zongsx@126.com

Supplementary Table S1. Detail known distribution data of *Euwallacea fornicatus*.

State	country	province/states	county	Latitude	Longitude	source
ASIA	Bangladesh	-	-	24.00	90.00	CABI
ASIA	Bon Islands	-	-	5.21	120.57	Literature
ASIA	Burma	-	-	21.91	95.96	Literature
ASIA	Cambodia	-	-	13.00	105.00	CABI
ASIA	India	Assam	-	26.00	93.00	CABI
ASIA	India	Karnataka	-	13.50	76.00	CABI
ASIA	India	Kerala	-	10.00	76.50	CABI
ASIA	India	Maharashtra	-	10.00	76.50	CABI
ASIA	India	Tamil Nadu	-	11.00	78.00	CABI
ASIA	India	Uttar Pradesh	-	27.25	80.75	CABI
ASIA	India	West Bengal	-	24.00	88.00	CABI
ASIA	Indonesia	Java	-	-7.61	110.72	CABI
ASIA	Indonesia	Kalimantan	-	0.96	114.56	CABI
ASIA	Indonesia	Sumatra	-	-0.59	101.35	CABI
ASIA	Indonesia	Borneo	-	0.96	114.55	Literature
ASIA	Israel	-	-	31.50	34.75	CABI
ASIA	Japan	Bonin Island	-	27.04	142.14	CABI
ASIA	Japan	Ryukyu Archipelago	-	26.50	128.01	CABI
ASIA	Laos	-	-	18.00	105.00	CABI
ASIA	Malaysia	Sarawak	-	2.50	113.50	CABI
ASIA	Malaysia	Peninsular Malaysia	-	4.42	114.00	CABI
ASIA	Malaysia	Sabah	-	5.50	117.00	CABI
ASIA	Myanmar	-	-	22.00	98.00	CABI
ASIA	Philippines	-	-	12.88	121.78	CABI
ASIA	Sri Lanka	-	-	7.87	80.78	CABI
ASIA	Vietnam	-	-	14.06	108.22	CABI
ASIA	Thailiand	Brunei Darussalam	-	4.54	114.73	Literature
AFRICA	Comoros	-	-	-12.17	44.25	CABI
AFRICA	Madagascar	-	-	-20.00	47.00	CABI
AFRICA	Réunion	-	-	-21.12	55.54	CABI
NORTH AMERICA	USA	California	Los Angeles	34.05	-118.24	Literature
NORTH AMERICA	USA	California	Orange Counties	33.72	-117.83	Literature
NORTH AMERICA	USA	California	San Bernardino	34.11	-117.29	Literature
NORTH AMERICA	USA	California	Ventura	34.27	-119.23	Literature
NORTH AMERICA	USA	California	San Diego	32.72	-117.16	Literature
NORTH AMERICA	USA	California	Riverside	-117.40	33.95	Literature
NORTH AMERICA	USA	California	Santa Barbara	-119.70	34.42	Literature
NORTH AMERICA	USA	Florida	Dade County	25.55	-80.63	Literature
NORTH AMERICA	USA	Hawaii	-	20.75	-156.50	CABI
CENTRAL AMERICA AND CARIBBEAN	Costa Rica	-	-	10.00	-84.00	CABI

CENTRAL AMERICA AND CARIBBEAN	Guatemala	-	-	15.50	-90.25	CABI
CENTRAL AMERICA AND CARIBBEAN	Panama	-	-	9.00	-80.00	CABI
OCEANIA	Australia	-	-	-16.00	167.00	Literature
OCEANIA	New Hebrides	-	-	-15.38	166.96	Literature
OCEANIA	Caroline Islands	–	-	6.05	147.08	Literature
OCEANIA	Fiji	-	-	-18.00	178.00	CABI
OCEANIA	Micronesia, Federated states of	-	-	6.89	158.22	CABI
OCEANIA	Niue	-	-	-19.05	-169.86	CABI
OCEANIA	Palau	-	-	7.51	134.59	CABI
OCEANIA	Papua New Guinea	-	-	-6.00	147.00	CABI
OCEANIA	Samoa	-	-	-13.58	-172.33	CABI
OCEANIA	Solomon Islands	-	-	-8.00	159.00	CABI
OCEANIA	Vanuatu	-	-	-16.00	167.00	CABI

Supplementary Table S2. Assessment results for the five groups of climate data in 12 months (compare the interpolated data to the observed data).

Month	Monthly average max. temperature			Monthly average min. temperature			Monthly average precipitation			Monthly average 9 am RH			Monthly average 3 pm RH		
	SE	R^2	P-value	SE	R^2	P-value	SE	R^2	P-value	SE	R^2	P-value	SE	R^2	P-value
1	2.21	0.97	<0.001	2.13	0.98	<0.001	5.68	0.98	<0.001	3.94	0.94	<0.001	3.39	0.94	<0.001
2	2.34	0.95	<0.001	2.18	0.97	<0.001	7.34	0.98	<0.001	3.76	0.96	<0.001	3.23	0.96	<0.001
3	2.42	0.92	<0.001	2.14	0.96	<0.001	9.66	0.98	<0.001	3.58	0.97	<0.001	3.08	0.97	<0.001
4	2.60	0.85	<0.001	2.17	0.94	<0.001	10.87	0.99	<0.001	3.39	0.98	<0.001	2.90	0.97	<0.001
5	2.70	0.80	<0.001	2.16	0.93	<0.001	22.95	0.96	<0.001	3.19	0.97	<0.001	2.72	0.97	<0.001
6	2.73	0.80	<0.001	2.11	0.92	<0.001	28.53	0.95	<0.001	3.31	0.96	<0.001	2.83	0.96	<0.001
7	2.69	0.82	<0.001	2.11	0.92	<0.001	29.73	0.91	<0.001	3.54	0.92	<0.001	3.04	0.91	<0.001
8	2.63	0.82	<0.001	2.12	0.93	<0.001	29.26	0.87	<0.001	3.34	0.91	<0.001	2.88	0.91	<0.001
9	2.55	0.85	<0.001	2.08	0.94	<0.001	18.38	0.91	<0.001	3.17	0.92	<0.001	2.77	0.92	<0.001
10	2.43	0.91	<0.001	2.02	0.96	<0.001	23.33	0.80	<0.001	3.13	0.94	<0.001	2.74	0.94	<0.001
11	2.21	0.96	<0.001	1.98	0.97	<0.001	6.86	0.96	<0.001	3.64	0.93	<0.001	3.16	0.93	<0.001
12	2.11	0.97	<0.001	2.04	0.98	<0.001	3.37	0.98	<0.001	4.04	0.92	<0.001	3.49	0.92	<0.001

Note: SE means Standard Error, RH means Relative Humidity.

Supplementary Table S3. Changes in the different parameters in parameters sensitivity analysis.

	DV0	DV1	DV2	DV3	SM0	SM1	SM2	SM3	TTCS	THCS	TTHS	THHS	SMDS	HDS	SMWS	HWS	PDD	EI_av
baseline	15	26	35	40	0.05	0.3	1	2.5	-10	-0.005	42	0.0002	0.05	-0.001	2.5	0.005	373	9.70
DV0-1	10	26	35	40	0.05	0.3	1	2.5	-10	-0.005	42	0.0002	0.05	-0.001	2.5	0.005	373	12.42
DV0-2	11	26	35	40	0.05	0.3	1	2.5	-10	-0.005	42	0.0002	0.05	-0.001	2.5	0.005	373	11.88
DV0-3	12	26	35	40	0.05	0.3	1	2.5	-10	-0.005	42	0.0002	0.05	-0.001	2.5	0.005	373	11.34
DV0-4	13	26	35	40	0.05	0.3	1	2.5	-10	-0.005	42	0.0002	0.05	-0.001	2.5	0.005	373	10.80
DV0-5	14	26	35	40	0.05	0.3	1	2.5	-10	-0.005	42	0.0002	0.05	-0.001	2.5	0.005	373	10.26
DV0-6	16	26	35	40	0.05	0.3	1	2.5	-10	-0.005	42	0.0002	0.05	-0.001	2.5	0.005	373	9.04
DV0-7	17	26	35	40	0.05	0.3	1	2.5	-10	-0.005	42	0.0002	0.05	-0.001	2.5	0.005	373	8.57
DV0-8	18	26	35	40	0.05	0.3	1	2.5	-10	-0.005	42	0.0002	0.05	-0.001	2.5	0.005	373	7.97
DV0-9	19	26	35	40	0.05	0.3	1	2.5	-10	-0.005	42	0.0002	0.05	-0.001	2.5	0.005	373	7.34
DV0-10	20	26	35	40	0.05	0.3	1	2.5	-10	-0.005	42	0.0002	0.05	-0.001	2.5	0.005	373	6.70
DV1-1	15	21	35	40	0.05	0.3	1	2.5	-10	-0.005	42	0.0002	0.05	-0.001	2.5	0.005	373	6.92
DV1-2	15	22	35	40	0.05	0.3	1	2.5	-10	-0.005	42	0.0002	0.05	-0.001	2.5	0.005	373	7.38
DV1-3	15	23	35	40	0.05	0.3	1	2.5	-10	-0.005	42	0.0002	0.05	-0.001	2.5	0.005	373	7.90
DV1-4	15	24	35	40	0.05	0.3	1	2.5	-10	-0.005	42	0.0002	0.05	-0.001	2.5	0.005	373	8.47
DV1-5	15	26	35	40	0.05	0.3	1	2.5	-10	-0.005	42	0.0002	0.05	-0.001	2.5	0.005	373	9.08
DV1-6	15	27	35	40	0.05	0.3	1	2.5	-10	-0.005	42	0.0002	0.05	-0.001	2.5	0.005	373	10.33
DV1-7	15	28	35	40	0.05	0.3	1	2.5	-10	-0.005	42	0.0002	0.05	-0.001	2.5	0.005	373	10.99
DV1-8	15	29	35	40	0.05	0.3	1	2.5	-10	-0.005	42	0.0002	0.05	-0.001	2.5	0.005	373	11.66
DV1-9	15	30	35	40	0.05	0.3	1	2.5	-10	-0.005	42	0.0002	0.05	-0.001	2.5	0.005	373	12.36
DV1-10	15	31	35	40	0.05	0.3	1	2.5	-10	-0.005	42	0.0002	0.05	-0.001	2.5	0.005	373	13.08
DV2-1	15	26	30	40	0.05	0.3	1	2.5	-10	-0.005	42	0.0002	0.05	-0.001	2.5	0.005	373	9.24
DV2-2	15	26	31	40	0.05	0.3	1	2.5	-10	-0.005	42	0.0002	0.05	-0.001	2.5	0.005	373	9.48
DV2-3	15	26	32	40	0.05	0.3	1	2.5	-10	-0.005	42	0.0002	0.05	-0.001	2.5	0.005	373	9.62
DV2-4	15	26	33	40	0.05	0.3	1	2.5	-10	-0.005	42	0.0002	0.05	-0.001	2.5	0.005	373	9.68

DV2-5	15	26	34	40	0.05	0.3	1	2.5	-10	-0.005	42	0.0002	0.05	-0.001	2.5	0.005	373	9.70
DV2-6	15	26	36	40	0.05	0.3	1	2.5	-10	-0.005	42	0.0002	0.05	-0.001	2.5	0.005	373	9.70
DV2-7	15	26	37	40	0.05	0.3	1	2.5	-10	-0.005	42	0.0002	0.05	-0.001	2.5	0.005	373	9.70
DV2-8	15	26	38	40	0.05	0.3	1	2.5	-10	-0.005	42	0.0002	0.05	-0.001	2.5	0.005	373	9.70
DV2-9	15	26	39	40	0.05	0.3	1	2.5	-10	-0.005	42	0.0002	0.05	-0.001	2.5	0.005	373	9.70
DV3-1	15	26	35	36	0.05	0.3	1	2.5	-10	-0.005	42	0.0002	0.05	-0.001	2.5	0.005	373	9.70
DV3-2	15	26	35	37	0.05	0.3	1	2.5	-10	-0.005	42	0.0002	0.05	-0.001	2.5	0.005	373	9.70
DV3-3	15	26	35	38	0.05	0.3	1	2.5	-10	-0.005	42	0.0002	0.05	-0.001	2.5	0.005	373	9.70
DV3-4	15	26	35	39	0.05	0.3	1	2.5	-10	-0.005	42	0.0002	0.05	-0.001	2.5	0.005	373	9.70
DV3-5	15	26	35	41	0.05	0.3	1	2.5	-10	-0.005	42	0.0002	0.05	-0.001	2.5	0.005	373	9.70
DV3-6	15	26	35	42	0.05	0.3	1	2.5	-10	-0.005	42	0.0002	0.05	-0.001	2.5	0.005	373	9.70
DV3-7	15	26	35	43	0.05	0.3	1	2.5	-10	-0.005	43	0.0002	0.05	-0.001	2.5	0.005	373	9.70
DV3-8	15	26	35	44	0.05	0.3	1	2.5	-10	-0.005	44	0.0002	0.05	-0.001	2.5	0.005	373	9.70
DV3-9	15	26	35	45	0.05	0.3	1	2.5	-10	-0.005	45	0.0002	0.05	-0.001	2.5	0.005	373	9.70
SM0-1	15	26	35	40	0	0.3	1	2.5	-10	-0.005	42	0.0002	0	-0.001	2.5	0.005	373	9.89
SM0-2	15	26	35	40	0.01	0.3	1	2.5	-10	-0.005	42	0.0002	0.01	-0.001	2.5	0.005	373	9.83
SM0-3	15	26	35	40	0.02	0.3	1	2.5	-10	-0.005	42	0.0002	0.02	-0.001	2.5	0.005	373	9.78
SM0-4	15	26	35	40	0.03	0.3	1	2.5	-10	-0.005	42	0.0002	0.03	-0.001	2.5	0.005	373	9.75
SM0-5	15	26	35	40	0.04	0.3	1	2.5	-10	-0.005	42	0.0002	0.04	-0.001	2.5	0.005	373	9.72
SM0-6	15	26	35	40	0.06	0.3	1	2.5	-10	-0.005	42	0.0002	0.05	-0.001	2.5	0.005	373	9.68
SM0-7	15	26	35	40	0.07	0.3	1	2.5	-10	-0.005	42	0.0002	0.05	-0.001	2.5	0.005	373	9.66
SM0-8	15	26	35	40	0.08	0.3	1	2.5	-10	-0.005	42	0.0002	0.05	-0.001	2.5	0.005	373	9.64
SM0-9	15	26	35	40	0.09	0.3	1	2.5	-10	-0.005	42	0.0002	0.05	-0.001	2.5	0.005	373	9.62
SM0-10	15	26	35	40	0.1	0.3	1	2.5	-10	-0.005	42	0.0002	0.05	-0.001	2.5	0.005	373	9.60
SM1-1	15	26	35	40	0.05	0.25	1	2.5	-10	-0.005	42	0.0002	0.05	-0.001	2.5	0.005	373	9.81
SM1-2	15	26	35	40	0.05	0.26	1	2.5	-10	-0.005	42	0.0002	0.05	-0.001	2.5	0.005	373	9.79
SM1-3	15	26	35	40	0.05	0.27	1	2.5	-10	-0.005	42	0.0002	0.05	-0.001	2.5	0.005	373	9.77

SM1-4	15	26	35	40	0.05	0.28	1	2.5	-10	-0.005	42	0.0002	0.05	-0.001	2.5	0.005	373	9.75
SM1-5	15	26	35	40	0.05	0.29	1	2.5	-10	-0.005	42	0.0002	0.05	-0.001	2.5	0.005	373	9.72
SM1-6	15	26	35	40	0.05	0.31	1	2.5	-10	-0.005	42	0.0002	0.05	-0.001	2.5	0.005	373	9.68
SM1-7	15	26	35	40	0.05	0.32	1	2.5	-10	-0.005	42	0.0002	0.05	-0.001	2.5	0.005	373	9.65
SM1-8	15	26	35	40	0.05	0.33	1	2.5	-10	-0.005	42	0.0002	0.05	-0.001	2.5	0.005	373	9.63
SM1-9	15	26	35	40	0.05	0.34	1	2.5	-10	-0.005	42	0.0002	0.05	-0.001	2.5	0.005	373	9.61
SM1-10	15	26	35	40	0.05	0.35	1	2.5	-10	-0.005	42	0.0002	0.05	-0.001	2.5	0.005	373	9.59
SM2-1	15	26	35	40	0.05	0.3	0.95	2.5	-10	-0.005	42	0.0002	0.05	-0.001	2.5	0.005	373	9.49
SM2-2	15	26	35	40	0.05	0.3	0.96	2.5	-10	-0.005	42	0.0002	0.05	-0.001	2.5	0.005	373	9.53
SM2-3	15	26	35	40	0.05	0.3	0.97	2.5	-10	-0.005	42	0.0002	0.05	-0.001	2.5	0.005	373	9.57
SM2-4	15	26	35	40	0.05	0.3	0.98	2.5	-10	-0.005	42	0.0002	0.05	-0.001	2.5	0.005	373	9.62
SM2-5	15	26	35	40	0.05	0.3	0.99	2.5	-10	-0.005	42	0.0002	0.05	-0.001	2.5	0.005	373	9.66
SM2-6	15	26	35	40	0.05	0.3	1.01	2.5	-10	-0.005	42	0.0002	0.05	-0.001	2.5	0.005	373	9.74
SM2-7	15	26	35	40	0.05	0.3	1.02	2.5	-10	-0.005	42	0.0002	0.05	-0.001	2.5	0.005	373	9.79
SM2-8	15	26	35	40	0.05	0.3	1.03	2.5	-10	-0.005	42	0.0002	0.05	-0.001	2.5	0.005	373	9.83
SM2-9	15	26	35	40	0.05	0.3	1.04	2.5	-10	-0.005	42	0.0002	0.05	-0.001	2.5	0.005	373	9.87
SM2-10	15	26	35	40	0.05	0.3	1.05	2.5	-10	-0.005	42	0.0002	0.05	-0.001	2.5	0.005	373	9.92
SM3-1	15	26	35	40	0.05	0.3	1	2.45	-10	-0.005	42	0.0002	0.05	-0.001	2.5	0.005	373	9.62
SM3-2	15	26	35	40	0.05	0.3	1	2.46	-10	-0.005	42	0.0002	0.05	-0.001	2.5	0.005	373	9.64
SM3-3	15	26	35	40	0.05	0.3	1	2.47	-10	-0.005	42	0.0002	0.05	-0.001	2.5	0.005	373	9.65
SM3-4	15	26	35	40	0.05	0.3	1	2.48	-10	-0.005	42	0.0002	0.05	-0.001	2.5	0.005	373	9.67
SM3-5	15	26	35	40	0.05	0.3	1	2.49	-10	-0.005	42	0.0002	0.05	-0.001	2.5	0.005	373	9.69
SM3-6	15	26	35	40	0.05	0.3	1	2.51	-10	-0.005	42	0.0002	0.05	-0.001	2.51	0.005	373	9.72
SM3-7	15	26	35	40	0.05	0.3	1	2.52	-10	-0.005	42	0.0002	0.05	-0.001	2.52	0.005	373	9.73
SM3-8	15	26	35	40	0.05	0.3	1	2.53	-10	-0.005	42	0.0002	0.05	-0.001	2.53	0.005	373	9.74
SM3-9	15	26	35	40	0.05	0.3	1	2.54	-10	-0.005	42	0.0002	0.05	-0.001	2.54	0.005	373	9.76
SM3-10	15	26	35	40	0.05	0.3	1	2.55	-10	-0.005	42	0.0002	0.05	-0.001	2.55	0.005	373	9.77

TTCS-1	15	26	35	40	0.05	0.3	1	2.5	-15	-0.005	42	0.0002	0.05	-0.001	2.5	0.005	373	10.43
TTCS-2	15	26	35	40	0.05	0.3	1	2.5	-14	-0.005	42	0.0002	0.05	-0.001	2.5	0.005	373	10.28
TTCS-3	15	26	35	40	0.05	0.3	1	2.5	-13	-0.005	42	0.0002	0.05	-0.001	2.5	0.005	373	10.13
TTCS-4	15	26	35	40	0.05	0.3	1	2.5	-12	-0.005	42	0.0002	0.05	-0.001	2.5	0.005	373	9.98
TTCS-5	15	26	35	40	0.05	0.3	1	2.5	-11	-0.005	42	0.0002	0.05	-0.001	2.5	0.005	373	9.83
TTCS-6	15	26	35	40	0.05	0.3	1	2.5	-9	-0.005	42	0.0002	0.05	-0.001	2.5	0.005	373	9.49
TTCS-7	15	26	35	40	0.05	0.3	1	2.5	-8	-0.005	42	0.0002	0.05	-0.001	2.5	0.005	373	9.38
TTCS-8	15	26	35	40	0.05	0.3	1	2.5	-7	-0.005	42	0.0002	0.05	-0.001	2.5	0.005	373	9.28
TTCS-9	15	26	35	40	0.05	0.3	1	2.5	-6	-0.005	42	0.0002	0.05	-0.001	2.5	0.005	373	9.16
TTCS-10	15	26	35	40	0.05	0.3	1	2.5	-10	-0.005	42	0.0002	0.05	-0.001	2.5	0.005	373	9.01
PDD-1	15	26	35	40	0.05	0.3	1	2.5	-10	-0.005	42	0.0002	0.05	-0.001	2.5	0.005	273	9.75
PDD-2	15	26	35	40	0.05	0.3	1	2.5	-10	-0.005	42	0.0002	0.05	-0.001	2.5	0.005	293	9.74
PDD-3	15	26	35	40	0.05	0.3	1	2.5	-10	-0.005	42	0.0002	0.05	-0.001	2.5	0.005	313	9.73
PDD-4	15	26	35	40	0.05	0.3	1	2.5	-10	-0.005	42	0.0002	0.05	-0.001	2.5	0.005	333	9.72
PDD-5	15	26	35	40	0.05	0.3	1	2.5	-10	-0.005	42	0.0002	0.05	-0.001	2.5	0.005	353	9.71
PDD-6	15	26	35	40	0.05	0.3	1	2.5	-10	-0.005	42	0.0002	0.05	-0.001	2.5	0.005	393	9.59
PDD-7	15	26	35	40	0.05	0.3	1	2.5	-10	-0.005	42	0.0002	0.05	-0.001	2.5	0.005	413	9.57
PDD-8	15	26	35	40	0.05	0.3	1	2.5	-10	-0.005	42	0.0002	0.05	-0.001	2.5	0.005	433	9.56
PDD-9	15	26	35	40	0.05	0.3	1	2.5	-10	-0.005	42	0.0002	0.05	-0.001	2.5	0.005	453	9.55
PDD-10	15	26	35	40	0.05	0.3	1	2.5	-10	-0.005	42	0.0002	0.05	-0.001	2.5	0.005	473	9.53

Note: EI_av means the average EI value of all the grids.

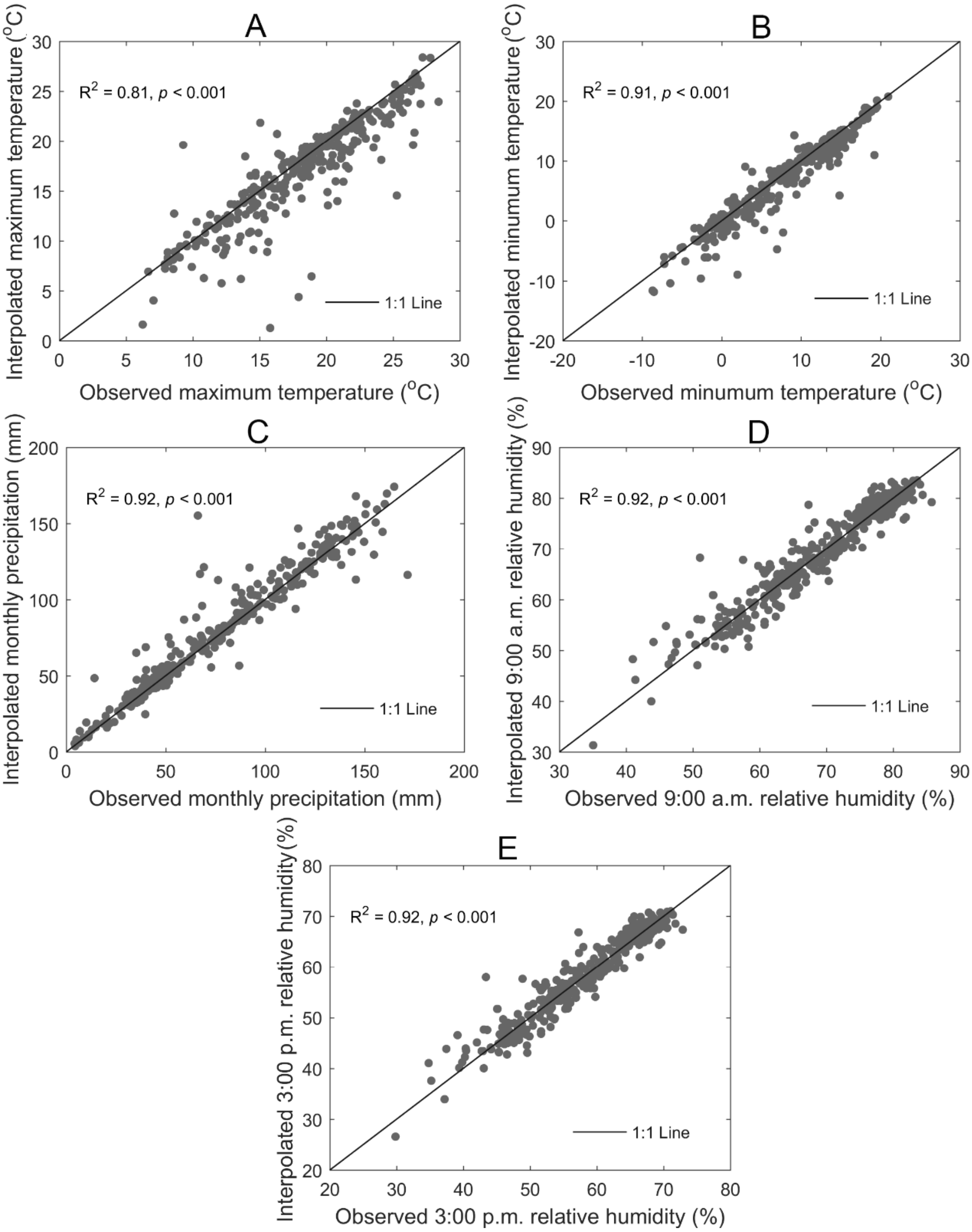

Supplementary Figure S1. Assessment results for the five groups of annual average climate data (compare the interpolated data to the observed data).
